# One-Step Synthesis of Bamboo Biochar for Efficiency Adsorption of Tetracycline: Characterization, Kinetics and Cost–Benefit Analysis

**DOI:** 10.3390/ma19071457

**Published:** 2026-04-05

**Authors:** Qi Liao, Chengyang Cao, Qiming Zhang, Pei Jia, Lu Dong

**Affiliations:** 1 School of Resource & Safety Engineering, Wuhan Institute of Technology, Wuhan 430074, China; w1980016406@163.com (Q.L.); zhangqiming2736@163.com (Q.Z.); 2 State Key Laboratory of Coal Combustion, Huazhong University of Science and Technology, Wuhan 430074, China; ludong@hust.edu.cn

**Keywords:** tetracycline removal, bamboo biochar, molten salt, adsorption kinetics, cost-effectiveness analysis

## Abstract

Tetracycline has been widely used as an efficient broad-spectrum antibiotic, while its long-term environmental pollution characteristics have gradually gained recognition and attention, highlighting the urgent need to identify a low-cost and effective method for removing tetracycline pollutants. This study aims to develop a one-step bamboo-based biochar preparation method based on a KCl-ZnCl_2_ molten salt system; the potential application of obtained bamboo-based biochar as a tetracycline adsorbent was characterized and analyzed. Results show that the biochar prepared at 900 °C possesses abundant microporous and mesoporous structures, with abundant surface functional groups. Also, it exhibits a composite type I/IV isotherm, with a specific surface area of 1305.91 m^2^·g^−1^, a total pore volume of 0.944 cm^3^·g^−1^, demonstrating excellent tetracycline adsorption capacity of 298.93 mg·g^−1^. XRD analysis confirmed that increasing the activation temperature significantly enhanced the graphitization degree of the biochar, which is a key factor influencing its tetracycline adsorption performance. Kinetic studies indicated that the adsorption kinetic process was better described by the Elovich model and Freundlich isotherm. Furthermore, cost-effectiveness analysis revealed that the cyclic preparation cost of biochar via this technique could be reduced to 18.25 USD per kilogram owing to the low consumption characteristics of the KCl-ZnCl_2_ molten salt, which represents a 93.4% reduction compared with conventional preparation methods, underscoring the economic applicability of this technology in the field of tetracycline removal. The findings of this study are expected to lay a foundation for the industrial preparation of low-cost, high-performance bamboo-based biochar for tetracycline removal.

## 1. Introduction

Tetracycline is currently one of the most widely used antibiotics worldwide [1]. Owing to its broad-spectrum antibacterial activity and good therapeutic efficacy, it has been extensively employed in medical, livestock, and poultry farming industries [2]. According to relevant statistics, the global consumption of antibiotics in food animals was 63,000 tons in 2015 and is projected to increase by nearly 70% by 2030. However, in recent years, tetracycline components have been detected in environmental water bodies such as wastewater treatment plants and drinking water in multiple countries, which draws severe attention to its recalcitrance to degradation and tendency to accumulate. Related studies have found that tetracycline not only exerts persistent ecological toxicity by influencing microbial community structure and disrupting inherent food chains in ecosystems, but also induces various antibiotic-resistant bacteria, and thus pose a global public safety risk [3]. Consequently, environmental pollution caused by tetracycline has garnered widespread concern and attention, and the World Health Organization has identified pollution from tetracycline and related antibiotics as one of the critical public health issues of the 21st century.

To address the need for treating and disposing of low-concentration tetracycline pollutants in environmental water bodies, numerous researchers have proposed various methods such as photocatalytic degradation [4], microbial degradation [5], and adsorptive removal [6]. Among these, the adsorptive removal method based on biochar has been extensively studied and applied due to its advantages of wide availability and low cost [7]. Several researchers [8,9,10,11,12] reviewed the application prospects of biochar derived from various biomass sources, including bamboo, poplar wood, and rice husk, in the field of tetracycline adsorption and removal. Their findings indicate that the pore size distribution of biochar is a critical factor influencing its tetracycline adsorption performance, and bamboo-based biochar exhibits a relatively high cost-effectiveness. Furthermore, Ru et al. employed ZnCl_2_ as an activating agent to prepare bamboo-based biochar with high adsorption performance for tetracycline removal through pyrolysis at 500 °C, increasing the maximum tetracycline adsorption capacity from 25.2 mg/g to 64.5 mg/g [13]. Meanwhile, Zhao et al. used KOH to produce biochar at 450 °C, which also demonstrated excellent tetracycline adsorption performance, elevating the maximum tetracycline adsorption capacity from 5.93 mg/g to 104.67 mg/g [14]. Notably, although the doping of ZnCl_2_ and KOH can enhance the tetracycline adsorption capacity of bamboo-based biochar, it also significantly increases the preparation cost, posing substantial obstacles to its application in the industrial field.

Numerous researchers have conducted extensive attempts to develop a low-cost, high-performance preparation method for bamboo-based biochar used in tetracycline removal. Piotr et al. found that zinc chloride and potassium chloride molten salts exhibit characteristics of low-loss rate and high-reaction rate during wood pyrolysis [15], indicating that chloride molten salts possess significant application potential in the field of bamboo-based biochar preparation. Meanwhile, the process parameters for biochar preparation are key factors influencing its adsorption performance. Studies by several researchers indicated that increasing the biochar concentration can effectively enhance the removal rate of tetracycline in water [16,17,18]. Research by Wasim et al. revealed that as the activation temperature increased from 600 °C to 800 °C, the microporous surface area steadily increased from 316 m^2^/g to 462 m^2^/g [19,20], contributing to improved adsorption performance of the biochar. Furthermore, studies by R. Puértolas et al. pointed out that pre-oxidation affects the subsequent pyrolysis of lignin; pre-oxidation induces structural changes in lignin and reduces its pyrolysis reactivity [21,22]. Therefore, establishing a bamboo-based biochar preparation technology based on a chloride molten salt system, combined with the optimization of process parameters, is expected to achieve the rapid preparation of low-cost bamboo-based biochar with high tetracycline adsorption performance.

Aimed at addressing the treatment and disposal needs for tetracycline pollution in environmental water bodies, this study utilized bamboo as the raw material and employed a KCl-ZnCl_2_ molten salt system to develop a one-step method for preparing bamboo-based biochar/activated carbon with high adsorption performance. The fundamental characteristics of the prepared bamboo-based biochar were characterized using techniques such as BET, FT-IR, SEM, and XRD. The effects of process parameters, including pre-oxidation temperature and activation temperature on tetracycline adsorption performance, were investigated. Adsorption kinetics and economic feasibility analyses were conducted to validate the application value of this research. The findings are expected to provide technical support for the industrial production of bamboo-based biochar with high adsorption efficiency and high economic value for tetracycline removal.

## 2. Materials and Methods

### 2.1. Experimental Materials

Bamboo was sourced from a bamboo plantation in Wuhan City, Hubei Province, China. The bamboo powder was dried in an oven at 105 °C for 6 h, followed by sieving through a 200-mesh sieve. Industrial activated carbon (IAC) was purchased from Henan Baide Water Purification Materials Co., Ltd. (Zhengzhou, China). Tetracycline (C_22_H_24_N_2_O_8_, 96%), zinc chloride (ZnCl_2_, 95%) and potassium chloride (KCl, 99.5%) were purchased from Shanghai Aladdin Biochemical Technology Co., Ltd. (Shanghai, China). Hydrochloric acid (HCl, 36–38%) was supplied by Xilong Scientific Co., Ltd. (Shantou, China). All solutions were prepared using ultrapure water (8.00 MΩ·cm).

### 2.2. Preparation of Biochar

In this study, a high-temperature melting method was employed to prepare KCl-ZnCl_2_ co-crystalline salt. Solid KCl and ZnCl_2_ were uniformly mixed at a mass ratio of 1.11:1, and the mixture was melted at 550 °C in a muffle furnace for 1 h. After cooling, the molten salt was ground into powder and stored under dry conditions.

Bamboo-based activated carbon was prepared via a one-step pyrolysis method. One gram of bamboo powder was placed in a crucible, covered with 10 g of KCl-ZnCl_2_ co-crystalline salt, and the crucible was then lidded. The material was heated from room temperature to 250 °C at a heating rate of 15 °C/min, held at 250 °C for 2 h, then further heated to 900 °C and maintained at this temperature for 1 h. After cooling, the activated carbon was collected. Deionized water in the amount of 100 mL was added to the activated carbon, and the mixture was stirred on a magnetic stirrer at 800 rpm for 12 h. Subsequently, ash was removed by washing with 0.1 mol/L diluted hydrochloric acid, followed by rinsing with deionized water until the filtrate reached neutral pH. The product was finally dried in an oven at 60 °C for 24 h to obtain the dried activated carbon.

### 2.3. Performance Characterization

#### 2.3.1. Characterization of Fundamental Parameters

Functional group information of the test samples was obtained through Fourier Transform Infrared Spectroscopy (FT-IR) analysis using a Nicolet 6700 spectrometer (Thermo Fischer, Waltham, MA, USA). The analysis was conducted under the following conditions: potassium bromide pellet method, wavenumber range of 400 to 4000 cm^−1^, resolution of 0.1 cm^−1^, and 32 scans. The pore structural parameters of the samples were characterized by nitrogen adsorption–desorption isotherms at 77 K using a Brunauer–Emmett–Teller (BET) surface area analyzer (Autosorb-iQ, Quantachrome, Boynton Beach, FL, USA). Prior to the analysis, samples were degassed at 200 °C for 8 h. The specific surface area was calculated using the BET method within the relative pressure (P/P_0_) range of 0.01 to 0.25. The microscopic morphology of the samples was observed by capturing Scanning Electron Microscopy (SEM) images using a tungsten filament scanning electron microscope (JSM-5510LV, JEOL Ltd., Tokyo, Japan).

The crystal structure was analyzed by X-ray Diffraction (XRD) using a D8 ADVANCE diffractometer (Bruker AXS GmbH, Karlsruhe, Germany) with Cu Kα radiation at 40 kV and 40 mA. Scans were performed with a step size of 0.017° over a 2θ range from 10° to 90°. The microcrystalline parameters for the 002 and 100 bands of the biochar and modified biochar, including the interlayer spacing (*d*_002_), stacking height (*L_c_*), and microcrystallite diameter (*L_a_*), could be calculated based on the Bragg and Scherrer equations.

(1) $$d_{002}=\frac{\lambda }{2sin\theta_{002}}$$

(2) $$L_{c}=\frac{0.89\lambda }{\beta_{002}cos\theta_{002}}$$

(3) $$L_{a}=\frac{1.84\lambda }{\beta_{100}\cos\theta_{100}}$$

Here, *λ* is the wavelength of copper radiation (0.15406 nm), *θ* is related to the peak position, and *β* is the corresponding full width at half maximum (FWHM).

#### 2.3.2. Characterization of Adsorption Performance

Batch adsorption experiments were conducted in a series of 250 mL volumetric flasks. A quantity of 0.01 g of biochar was mixed with 100 mL of tetracycline solution at a concentration of 50 mg/L. The suspension was placed in a thermostatic magnetic stirrer and agitated until the stirring duration reached 1 h. After 1 h, the concentration of residual tetracycline was determined using a UV-visible spectrophotometer. The adsorption capacity of the biochar for tetracycline was calculated according to the following formula:

(4) $$q_{e}=\frac{V\left(C_{o}-C_{e}\right)}{M}$$

In the equation, *q_e_* represents the adsorption capacity of tetracycline at adsorption equilibrium, with the unit of mg/g; *C*_0_ is the initial concentration of the tetracycline solution, with the unit of mg/L; *C_e_* is the equilibrium concentration of tetracycline, with the unit of mg/L; *V* is the volume of the solution, with the unit of L; and *M* is the mass of activated carbon used, with the unit of g.

Adsorption kinetics experiments were conducted to determine the kinetic behavior. Based on the adsorption experiments, investigations were carried out using tetracycline concentrations ranging from 20 to 80 mg/L over a time span of 0 to 360 min. The pseudo-first-order (PFO) kinetic model, pseudo-second-order (PSO) kinetic model, and Elovich model are presented in Equations (5)–(7), respectively.

(5) $$q_{t}=q_{e}\left(1-e^{-k_{1}t}\right)$$

(6) $$q_{t}=\frac{k_{2}{q_{e}}^{2}t}{1+k_{2}q_{e}t}$$

(7) $$q_{t}=\frac{1}{\beta }ln\left(1+a\beta t\right)$$

(8) $$q_{t}=k_{i}t^{1/2}+C$$

Nonlinear regression analysis employing the Gauss-Newton method was used to determine which of the PFO, PSO, or Elovich models best described the adsorption of tetracycline onto the biochar. The data were subsequently plotted on a linear scale to identify the presence of multilinearity in the intra-particle diffusion model, thereby determining whether multiple adsorption mechanisms existed. Here, *q_t_* (mg/g) is the adsorption capacity at time t (minutes), *q_e_* (mg/g) is the adsorption capacity at equilibrium, *k*_1_ is the pseudo-first-order rate constant (min^−1^), *k*_2_ is the pseudo-second-order rate constant (g·mg^−1^·min^−1^), *β* (g·mg^−1^) is the surface coverage and activation energy constant, *α* (mg·g^−1^·min^−1^) is the initial adsorption rate, *k_i_* (mg·g^−1^·min^−1/2^) is the rate constant for the intra-particle diffusion model, and *C* (mg/g) is the intercept related to the amount of tetracycline removed during the rapid initial adsorption stage.

The isotherm models analyzed in the adsorption isotherm experiments were the Langmuir, Freundlich and Sips models, as shown in Equations (9)–(11) respectively. Gauss–Newton nonlinear regression was performed on these equations to determine which model best described the isothermal behavior of tetracycline adsorption onto the biochar.

(9) $$q_{e}=\frac{C_{e}k_{L}q_{m}}{1+k_{L}C_{e}}$$

(10) $$q_{e}={k_{f}C_{e}}^{1/n}$$

(11) $$q_{e}=\frac{k_{s}q_{m}{C_{e}}^{s}}{1+k_{s}{C_{e}}^{s}}$$

where q_e_ (mg/g) is the adsorption at equilibrium, C_e_ (mg/L) is the concentration at equilibrium, k_L_ (l mg^−1^) is the Langmuir constant, q_m_ (mg/g) is the maximum adsorption capacity, k_f_ (1 g^−1^) is the Freundlich constant, *n* is the Freundlich constant that describes the intensity of adsorption to the biochar surface, k_s_ is the Sips adsorption constant, and s describes the surface heterogeneity as in the Freundlich model.

### 2.4. Cost-Effectiveness Analysis of Biochar

The price of biochar is attributed to variations in raw material costs and manufacturing processes, including collection and pretreatment, chemical agents, pyrolysis electricity, pyrolysis temperature, and other factors. In European and American markets, the price of biochar has decreased significantly from 2.74 USD per kilogram in 2013 to 0.6–1.3 USD per kilogram in 2021. Here, the price of biochar is based on the Chinese market and typically depends on the costs of various raw materials. This study only calculated the cost of biochar as well as the expenses for reagents and pyrolysis electricity, potentially underestimating the actual cost of the modified biochar technology. For the benefit of tetracycline removal, the cost-effectiveness of the biochar method is calculated using the following formula:

(12) $$Cost=\frac{1000\;\times \left(P_{BC}+P_{Acro}\right)\times {\;D}_{BC}}{C_{TC}\times {\;R}_{TC}}$$

where Cost refers to the expense required to remove 1 kg of tetracycline from wastewater (USD·kg^−1^), *D_BC_* is the dosage of biochar or modified biochar (g·L^−1^), *P_BC_* is the price per kilogram of biochar or modified biochar (USD·kg^−1^), *P_Acro_* is the electricity cost per kilogram of biochar or modified biochar (USD·kg^−1^), *C_TC_* is the initial tetracycline concentration (mg·L^−1^), *R_TC_* is the tetracycline removal rate (%), and 1000 is the unit conversion factor.

## 3. Results and Discussion

### 3.1. Characterization of Biochar Materials

#### 3.1.1. Fourier Transform Infrared Spectroscopy

**Figure 1:**
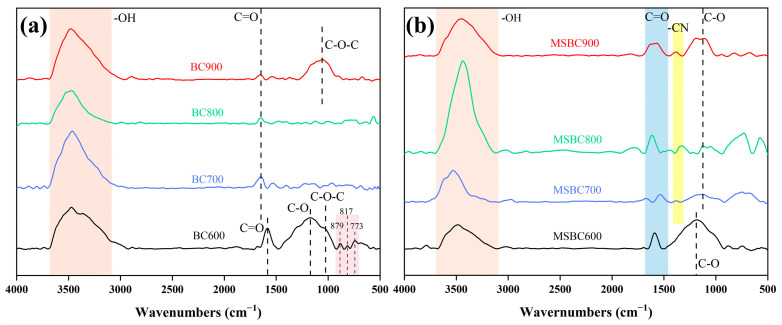
FT-IR of carbon: (**a**) Biochar prepared at 600–900 °C, (**b**) Modified biochar prepared at 600–900 °C.

The FTIR spectra of biochar (BC) and modified biochar (MSBC) synthesized from bamboo powder at activation temperatures ranging from 600 °C to 900 °C are illustrated in Figure 1. As can be observed from Figure 1a, as the activation temperature increases from 600 °C to 900 °C, the number of surface functional groups on the biochar decreases significantly. This is primarily due to the dissociation, decomposition, or transformation of surface functional groups such as hydroxyl, carbonyl, and C–O bonds during the carbonization of bamboo powder. In particular, samples at different temperatures exhibit a broad peak near the wavelength range of 3104–3685 cm^−1^, corresponding to hydroxyl stretching vibrations [23], which is associated with the rapid adsorption of moisture from the air by the rich porous structure of the biochar. Correspondingly, biochar samples prepared at an activation temperature of 600 °C show absorption peaks at 1583 cm^−1^, 1155 cm^−1^, and 1045 cm^−1^, corresponding to the vibrations of carbonyl, C–O bonds, and ether bonds, respectively. As the activation temperature increases, the corresponding absorption peaks gradually disappear, indicating the gradual decomposition or transformation of oxygen-containing functional groups during the carbonization process. Three absorption peaks are observed in the range of 900–650 cm^−1^, located at 879 cm^−1^, 817 cm^−1^, and 773 cm^−1^, corresponding to the out-of-plane bending of C–H bonds in aromatic rings, which suggests the presence of some aromatic compounds in BC600 [24].

Samples measured at different temperatures present a broad peak in the wavenumber range of 3147–3668 cm^−1^ associated with hydroxyl stretching vibrations, as presented in Figure 1b. Additionally, multiple absorption peaks are observed at 1565 cm^−1^ and 1189 cm^−1^, which correspond to the vibrations of carbonyl groups and C–O bonds, respectively. As the activation temperature increases, the intensity of the corresponding absorption peaks gradually weakens. Evidently, the number of surface functional groups on the biochar prepared under the molten salt system is significantly higher compared to that from conventional pyrolysis. Moreover, as the activation temperature increases, the number of surface functional groups remains stable, which also provides more chemical adsorption sites for the prepared biochar [25], thereby demonstrating superior application potential. At the same activation temperature, the absorption peak intensities for carbonyl groups and C–O bonds in the modified biochar are higher than those in the biochar, indicating that the modified biochar retains more oxygen-containing functional groups. This may be related to the inhibition of the transformation of some oxygen-containing functional groups by the KCl-ZnCl_2_ salt. Modified biochar samples prepared at activation temperatures of 700–900 °C show a relatively weak absorption peak at 1396 cm^−1^, corresponding to the vibration of the carbon–nitrogen bond in amides [26]. This is attributed to the Lewis acid catalysis by the KCl-ZnCl_2_ salt, which promotes dehydration reactions in cellulose, hemicellulose, lignin, and other components. Combined with high temperature, this leads to the cleavage of molecular chains in cellulose and hemicellulose. The resulting unsaturated short chains undergo aromatic condensation reactions with phenolic derivatives produced from lignin pyrolysis, forming thermally stable aromatic and heterocyclic aromatic compounds. It is noteworthy that reactions such as dehydration and aromatic condensation also lead to structural changes in the modified biochar, which is crucial for influencing its pore structure.

#### 3.1.2. BET

**Figure 2:**
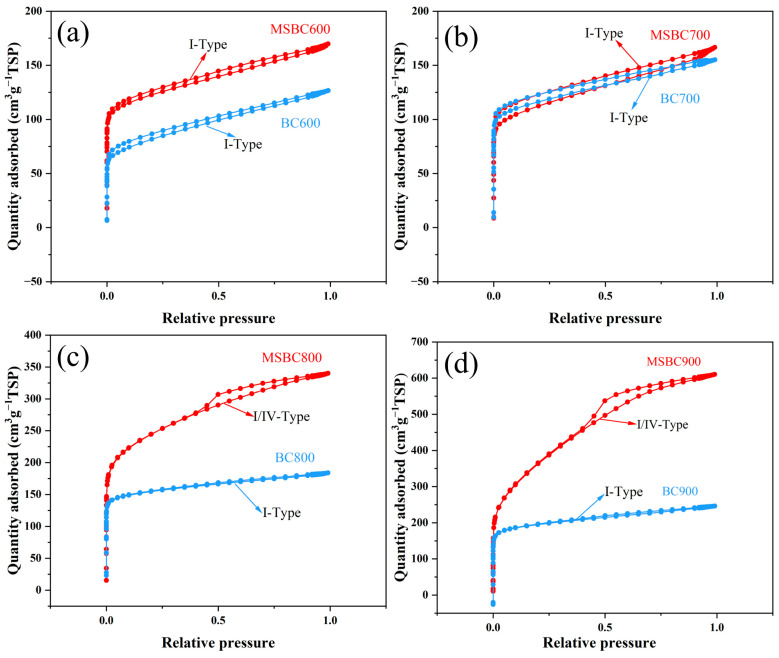
N_2_ adsorption–desorption isotherm: (**a**) BC600 and MSBC600, (**b**) BC700 and MSBC700, (**c**) BC800 and MSBC800, (**d**) BC900 and MSBC900.

The nitrogen adsorption–desorption isotherms of biochar (BC) and modified biochar (MSBC), derived from bamboo powder at activation temperatures of 600 °C to 900 °C, are presented in Figure 2. As can be observed from the N_2_ adsorption and desorption isotherms of the samples in Figure 2, the adsorption capacity of the modified biochar is higher than that of the biochar at the same activation temperature, and the adsorption capacity gradually increases with rising activation temperature. From Figure 2a,b, it can be seen that both the unmodified and modified biochars activated at 600 °C and 700 °C exhibit typical type I isotherms, indicating that these materials are primarily composed of micropores with a small number of mesopores, which is attributed to the relatively low activation temperature. From Figure 2c,d, it is observed that samples MSBC800 and MSBC900 exhibit typical type IV isotherms with H5-type hysteresis loops within the relative pressure (P/P_0_) range of 0.4–0.93, suggesting that these two materials consist mainly of mesopores and micropores [27]. The presence of the H5-type hysteresis loop in the samples indicates the existence of interconnected pores. This interconnected pore structure may be related to constrictions or “ink-bottle” pores within the pores, leading to a delay in the desorption process compared to the adsorption process. The absence of relative pressure shifts in the hysteresis loops of these two samples, along with an overall upward shift in the MSBC900 isotherm, suggests that both materials have similar mesopore size distributions, and the pore volume of MSBC900 is larger than that of MSBC800. Therefore, the modified biochar possesses a richer pore structure compared to the biochar. The molten salt system creates an environment of free zinc ions, potassium ions, and chloride ions, which facilitates the formation of mesopores within the material. Furthermore, the enhanced heat transfer provided by the molten salt system contributes to an increased rate of mesopore formation, and raising the activation temperature helps to increase the pore volume of the material.

The corresponding specific surface area, total pore volume, micropore surface area, and average pore diameter are summarized in Table 1. As can be seen from Table 1, the parameters of surface area, total pore volume, micropore surface area, and average pore diameter for the modified activated carbon are all higher than those of the unmodified biochar at the same activation temperature. Specifically, the specific surface area of MSBC600, prepared at a relatively low temperature, is 458.01 m^2^·g^−1^, which is 1.57 times that of the biochar (291.81 m^2^·g^−1^) prepared at the same temperature. As the activation temperature increases, the specific surface area of MSBC900 reaches 1305.91 m^2^·g^−1^, while the ratio of the specific surface area between the modified activated carbon and the biochar at the same temperature remains stable at approximately 1.49 times. As the activation temperature rises from 600 °C to 900 °C, the ratio of total pore volume between the modified biochar and the biochar increases from 1.34 to 2.21. This is attributed to the catalytic dehydration reactions and etching effects induced by zinc ions, potassium ions, and chloride ions on the bamboo powder during pyrolysis, combined with the enhanced heat transfer of the molten salt system, which synergistically promotes pore development in the biochar, leading to a gradual increase in its pore parameters. After modification with KCl-ZnCl_2_ salt, the biochar sample BC900 shows an increase in micropore volume from 0.335 cm^3^·g^−1^ to 0.430 cm^3^·g^−1^ (a ratio of 1.28 times) and in total pore volume from 0.428 cm^3^·g^−1^ to 0.944 cm^3^·g^−1^ (a ratio of 2.11 times). The percentage of micropore volume relative to the total volume decreases from 78.27% to 45.55%, and the average pore diameter increases from 0.86 nm to 1.46 nm. This is related to the characteristic of KCl-ZnCl_2_ salt in forming broad micropores and mesopores during the activation process, indicating that KCl-ZnCl_2_ salt modification facilitates the development of micropores into mesopores within the material. For the modified biochar sample MSBC600, when the activation temperature is increased to 900 °C (MSBC900), the micropore volume rises from 0.177 cm^3^·g^−1^ to 0.430 cm^3^·g^−1^ (a ratio of 2.43 times), and the total pore volume increases from 0.263 cm^3^·g^−1^ to 0.944 cm^3^·g^−1^ (a ratio of 3.39 times). The percentage of micropore volume relative to the total volume decreases from 67.30% to 45.55%, and the average pore diameter increases from 0.96 nm to 1.46 nm. This may be attributed to the increased catalytic rate of the KCl-ZnCl_2_ molten salt system at elevated temperatures, which facilitates the release of hydrogen during the decomposition of precursor phenol-aromatic structures, leading to the formation of more micropores.

#### 3.1.3. XRD

**Figure 3:**
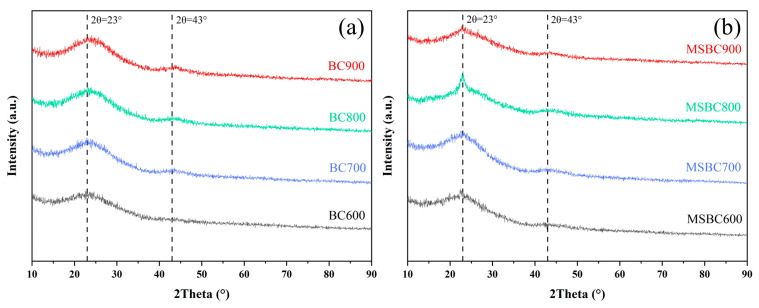
XRD patterns: (**a**) Biochar prepared at 600–900 °C, (**b**) Modified biochar prepared at 600–900 °C.

The X-ray diffraction (XRD) patterns of biochar (BC) and modified biochar (MSBC) fabricated from bamboo powder at activation temperatures between 600 °C and 900 °C are illustrated in Figure 3. As shown in Figure 3a,b, the patterns exhibit broad, low-intensity diffraction peaks near 2θ = 23° and 2θ = 43°, which are typically associated with the (002) and (100) crystal planes of turbostratic or partially graphitic structures. This indicates the presence of some disordered graphitic planes within the material, possessing a low degree of graphitization, and confirms that it is not entirely amorphous carbon. The peak position of the (002) band for both the biochar and modified biochar is around 23–24°, which is lower than the graphite peak (26.6°), suggesting that neither the biochar nor the modified biochar prepared at different activation temperatures has formed a graphite crystal structure [28].

From Figure 3a, it can be observed that as the activation temperature increases from 600 °C to 900 °C, the intensity of the diffraction peak at 2θ = 23° for the biochar samples gradually increases, indicating that higher activation temperatures promote the transformation of biochar towards graphitic microcrystals. This is likely due to the fact that elevated temperatures favor the dehydrogenation of aromatic clusters, allowing them to grow into larger graphene-like sheets through dehydrocondensation, suggesting that higher activation temperatures facilitate the transition of amorphous carbon from a disordered structure to a more ordered one. From Figure 3b, it can be seen that as the activation temperature rises from 600 °C to 900 °C, the intensity of the diffraction peak at 2θ = 23° for the modified biochar samples first increases and then decreases. This is because, at lower temperatures, the KCl-ZnCl_2_ salt promotes dehydration reactions, which, combined with the increased activation temperature, facilitates the graphitization of amorphous carbon, leading to an increase in diffraction peak intensity. At higher activation temperatures, the etching effect of the KCl-ZnCl_2_ salt disrupts the transformation of the modified biochar into graphitic microcrystals, causing the diffraction peak intensity to gradually decrease. Among the samples, MSBC800 exhibits a higher and sharper diffraction peak at 2θ = 23°, indicating a partially ordered stacking of the internal structure of the amorphous carbon, which most closely approximates graphitic microcrystals [29].

The microcrystalline parameters calculated from the XRD patterns of the carbon materials are listed in Table S1. As shown in Table S1, the *d*_002_ values of biochar BC600 and BC800 are 0.3862 nm and 0.3741 nm, respectively. This is primarily because, at low temperatures below 600 °C, the pyrolysis of biomass precursors forms highly disordered amorphous carbon, with interlayer spacing significantly larger than that of ideal graphite (0.335 nm). As the activation temperature increases, the interlayer spacing generally exhibits a decreasing trend, indicating that the carbon structure tends to become more ordered and graphitized at higher activation temperatures. The average stacking height (*Lc*) of biochar BC600 and BC800 is 0.0134 nm and 0.0152 nm, respectively, showing an overall increasing trend. This suggests that higher activation temperatures lead to an increase in-plane microcrystal size and a reduction in reactivity. As the activation temperature rises from 600 °C to 900 °C, the microcrystal diameter (*Lα*) increases from 0.0063 nm to 0.1052 nm. This indicates that increasing the activation temperature enhances the graphitization degree of the biochar, facilitates the formation of π-π stacking, and reduces the reactivity of the biochar [30].

As shown in Table S1, with increasing activation temperature, the interlayer spacing of modified biochar exhibits a trend of first decreasing and then increasing, indicating that both the KCl-ZnCl_2_ salt and the activation temperature jointly influence the interlayer spacing. The average *d*_002_ of modified biochar is approximately 0.389 nm, which is larger than the average interlayer spacing of biochar (0.382 nm), suggesting that the graphitization degree of modified biochar is lower than that of biochar. This is related to the richer functional group structure of modified biochar. The average stacking height of modified biochar first increases and then decreases with rising activation temperature, which is the result of the combined effects of zinc chloride, potassium chloride, and activation temperature. As the activation temperature increases, the microcrystal diameter of modified biochar increases from 0.0066 nm to 0.0815 nm, indicating that activated carbon prepared at higher temperatures exhibits greater carbon layer planar extensibility. The benzene ring in tetracycline interacts with the polarized aromatic rings of the graphitic structure in biochar through π-π electron donor–acceptor (EDA) interactions, and the protonated amino groups of tetracycline interact with biochar via cation-π bonding with the graphite π-electrons [31,32,33]. Therefore, enhancing the graphitization degree of biochar contributes to improving its adsorption performance for tetracycline.

#### 3.1.4. Tungsten Filament Scanning Electron Microscopy

**Figure 4:**
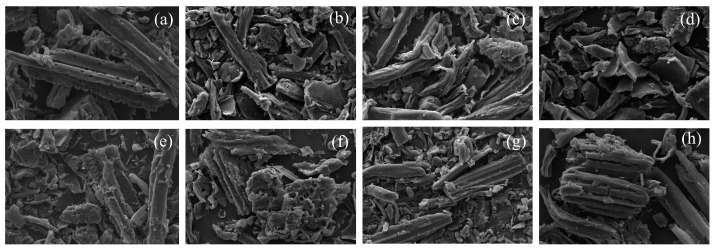
SEM images of modified biochar and biochar: (**a**) BC600, (**b**) BC700, (**c**) BC800, (**d**) BC900, (**e**) MSBC600, (**f**) MSBC700, (**g**) MSBC800, (**h**) MSBC900.

Scanning electron microscopy images of biochar and modified biochar derived from bamboo powder at activation temperatures ranging from 600 °C to 900 °C are exhibited in Figure 4. Figure 4a–d show the SEM images of biochar prepared at activation temperatures from 600 °C to 900 °C, respectively. The porous structure and fissures of the resulting biochar can be clearly observed in the SEM images. The image of sample BC600 reveals that the biochar primarily exists in rod-like forms with a small proportion of fragmented carbon. Its surface exhibits an irregular porous structure, predominantly consisting of macropores, which is attributed to the generation of volatile substances during pyrolysis that contribute to the formation of the biochar’s porous framework. The formation of rod-like carbon is likely associated with the thick-walled and highly lignified fiber cells that constitute the vascular bundle structure of bamboo, where the stable polycyclic aromatic structures formed during pyrolysis maintain the basic outline of the cells. The image of sample BC800 shows that the biochar mainly exists as shorter rod-like carbon, with an increased proportion of fragmented carbon. This may be related to structural reorganization of the biochar under high-temperature conditions, as increasing the activation temperature significantly influences the macro-structure. As the activation temperature rises from 600 °C to 900 °C, the biochar gradually develops a more abundant porous structure, indicating that higher activation temperatures accelerate the escape of substances, thereby facilitating the formation of a porous framework. Figure 4d presents the SEM image of biochar prepared at an activation temperature of 900 °C, revealing that the biochar primarily exists as fragmented carbon, with an increased number and size of macropores on its surface [34]. This suggests that higher activation temperatures lead to the generation of more fragmented carbon. This phenomenon is related to the characteristics of bamboo parenchyma cells, which possess high volatile content, high pore cavity volume, high thermal stress sensitivity, and low lignin content, making them prone to fragmentation during high-temperature pyrolysis. During the carbonization process, the original biomass material is transformed into carbon with a dense structure and underdeveloped porosity. The residual carbon material is arranged irregularly, leaving free volumes that may be occupied by amorphous carbon, leading to shrinkage or collapse of the carbon skeleton [35]. This indicates that higher activation temperatures exacerbate the shrinkage and collapse of the amorphous carbon skeleton, resulting in the formation of irregular biochar structures, and thereby reducing the uniformity of its pore structure.

The SEM images of modified biochar fabricated via the molten salt system at activation temperatures from 600 °C to 900 °C, which feature self-supporting and highly porous architectures, are depicted in Figure 4e–h. Specifically, the image of sample MSBC600 shows that the modified biochar primarily exists in rod-like carbon forms, and its surface possesses a more abundant three-dimensional porous structure compared to BC600. In contrast to the biochar obtained via direct pyrolysis, the biochar produced under the KCl-ZnCl_2_ salt mixture exhibits a well-developed three-dimensional architecture. The image of sample MSBC800 reveals carbon structures with interconnected interlayer pore networks resembling stacked flakes. This is primarily attributed to the infiltration of the KCl-ZnCl_2_ salt mixture into the carbon structure during pyrolysis, which prevents volumetric shrinkage caused by the compaction or collapse of the pore system and promotes the formation of high-quality porous carbon, as illustrated in Figure S1. During pyrolysis, the KCl-ZnCl_2_ salt accelerates dehydration reactions, and the salt molecules and their hydrates remaining inside the carbon particles act as molecular templates for the formation of micropores. Figure 4h is the SEM image of sample MSBC900, showing fissures on the surface of the tubular carbon, indicating that increased activation temperature reduces the stability of the tubular carbon, which may affect the overall adsorption performance of the modified biochar.

### 3.2. Effect of Process Parameters on Adsorption Performance

Many researchers have already recognized that process parameters significantly influence the adsorption of tetracycline onto biochar. Studies have indicated that adsorbent concentration affects tetracycline adsorption by altering the number of available adsorption sites; an increase in pyrolysis temperature facilitates the development of pore structures; pre-oxidation treatment helps retain more active sites during pyrolysis. Both pyrolysis temperature and pre-oxidation treatment impact the tetracycline adsorption performance of biochar by influencing liquid film diffusion and intraparticle diffusion. Therefore, conducting systematic and comprehensive investigations into process parameters is crucial for understanding their effects on tetracycline adsorption.

#### 3.2.1. Biochar Concentration

The adsorption-removal profile of adsorbent dosage against a tetracycline aqueous solution with an initial concentration of 50 mg/L is displayed in Figure S2. As the concentration of MSBC900 increased from 50 mg/L to 200 mg/L, the adsorption capacity per unit mass of modified biochar decreased from 353.90 mg/g to 210.37 mg/g, while the tetracycline removal efficiency increased from 32.13% to 87.72%. This is attributed to the excessive adsorption sites provided by the surplus adsorbent, leading to competitive adsorption among the sites for tetracycline and resulting in a high ratio of tetracycline molecules to available vacant sites. This indicates that an excessive amount of adsorbent can effectively remove tetracycline from the solution.

#### 3.2.2. Activation Temperature

Activation temperature is the most critical factor influencing the adsorption performance of modified biochar. Figure S3 illustrates the adsorption capacities of tetracycline by biochar and modified biochar prepared at activation temperatures ranging from 600 °C to 900 °C. As shown in Figure S3, the tetracycline adsorption capacities of biochar samples BC600, BC700, BC800, and BC900 are 22.51 mg/g, 21.36 mg/g, 20.10 mg/g, and 28.04 mg/g, respectively. The average tetracycline adsorption capacity of these four biochar samples is similar, with a mean value of 23.00 mg/g, indicating that increasing the activation temperature within the range of 600 °C to 900 °C has a minimal impact on enhancing the tetracycline adsorption performance of the biochar. This is attributed to its lack of abundant functional group structures, relatively small specific surface area, and the presence of fragmented carbon. This suggests that higher activation temperatures lead to the generation of fragmented carbon, which reduces the overall tetracycline adsorption performance of the biochar.

As observed in Figure S3, the tetracycline adsorption capacities of modified biochar samples MSBC600, MSBC700, MSBC800, and MSBC900 are 44.74 mg/g, 53.74 mg/g, 171.94 mg/g, and 298.93 mg/g, respectively. The adsorption capacity of the modified biochar is significantly enhanced compared to that of the biochar and exhibits a positive correlation with temperature. With increasing activation temperature, the adsorption capacities of the four modified biochar samples are 1.99, 2.52, 2.67, and 10.66 times higher than those of the biochar at the same temperatures, respectively, indicating that increasing the activation temperature within the molten salt system enables a leap-like improvement in the adsorption performance of the modified biochar.

The structure-activity relationship plots between the adsorption capacity of the modified biochar and its specific surface area, total pore volume, micropore surface area, and average pore diameter are displayed in Figures S4a, S4b, S4c, and S4d, respectively. The structure-activity relationships between the modified biochar and its porosity properties exhibit good linear correlations, with linear correlation coefficients (R^2^) all greater than 0.975. It can be concluded that micropore filling is one of the primary mechanisms for tetracycline adsorption by the modified biochar, and this relationship can be used to predict the adsorption capacity based on pore parameters within this range. This indicates that increasing the activation temperature influences adsorption performance by altering the pore size, pore structure, as well as the types and quantities of functional groups in the carbon material.

#### 3.2.3. Pre-Oxidation Temperature

The activation temperature during the pre-oxidation process is an important factor influencing the adsorption performance of modified biochar. The tetracycline adsorption capacities of modified activated carbon derived from bamboo powder with and without pre-oxidation at 200–300 °C prior to activation at 900 °C are illustrated in Figure S5. The results indicate that the adsorption capacities of modified biochar subjected to pre-oxidation at 200 °C, 250 °C, and 300 °C are 299.15 mg/g, 298.93 mg/g, and 290.98 mg/g, respectively. The adsorption capacity of modified biochar without pre-oxidation is 117.55 mg/g, with the ratio of adsorption capacity between pre-oxidized and non-pre-oxidized modified biochar being 2.52. This demonstrates that the pre-oxidation process enhances the adsorption performance of modified biochar, and bamboo exhibits a broad pre-oxidation temperature range, which is beneficial for improving the stability of its adsorption performance. During pre-oxidation at 250 °C, the intensities of carbon-oxygen bonds and carbonyl groups in the fingerprint region increase [36], hydroxyl groups in lignin are converted into carbonyl groups [37], and various types of lignin inter-chain bonds are cleaved. The pre-oxidation process likely helps the molten salt system retain more oxygen-containing functional groups on the modified biochar, and these active sites enhance the adsorption of tetracycline through van der Waals forces or π-π interactions.

### 3.3. Adsorption Kinetics

The objective of kinetic studies is to describe the adsorption rate of tetracycline onto biochar. Kinetic parameters aid in predicting the adsorption rate and provide essential information for process modeling. Adsorption mechanism studies were conducted to determine the rate-controlling step. Consequently, the intraparticle diffusion model was compared with the experimental data.

**Figure 5:**
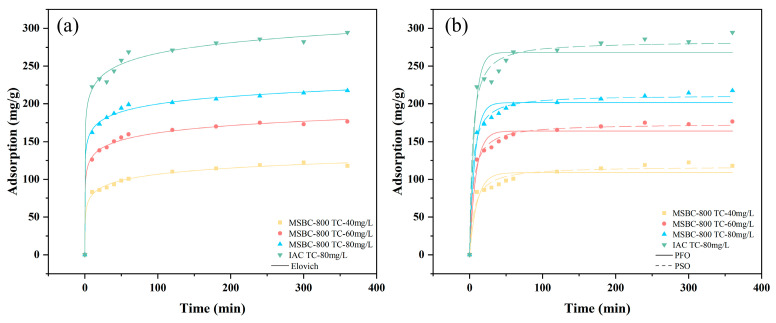
Experimental Kinetic date plotted with Elovich model (**a**), and PFO and PSO date (**b**).

The adsorption kinetic results presented in Figure 5 and Table 2 indicate that the order of fit for the kinetic models is Elovich > PSO > PFO. The Elovich model assumes that the surface of the biochar adsorbent is heterogeneous, implying that some parts of the surface possess a higher affinity for tetracycline adsorption than others [38]. The fitting of tetracycline adsorption using the Elovich model suggests that tetracycline binds tightly to the biochar, and thus the adsorbed tetracycline becomes immobilized. Furthermore, a general trend can be observed wherein the β value decreases with increasing initial concentration, indicating that more adsorption sites are utilized at higher initial concentrations. The PFO model describes the liquid film diffusion of tetracycline between the aqueous solution and the biochar surface [39].

**Figure 6:**
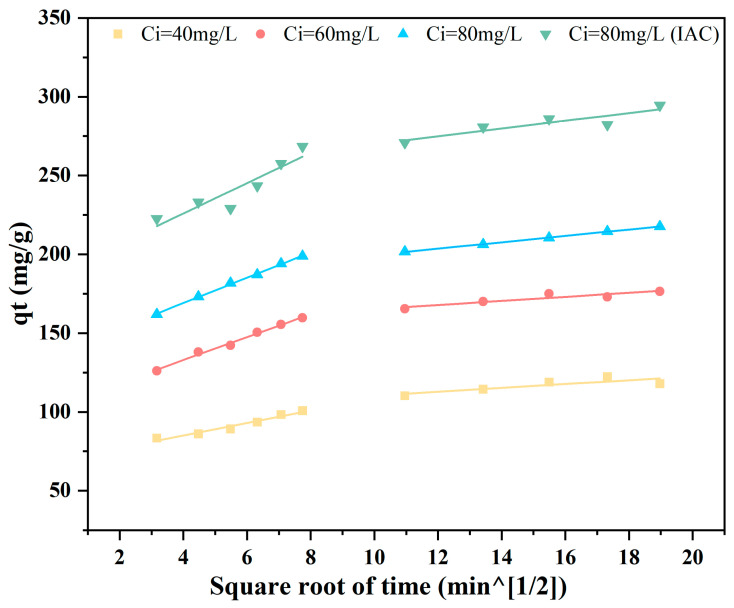
Intraparticle diffusion model for the adsorption of tetracycline on biochar.

The linear fitting plot of the intraparticle diffusion model is presented in Figure 6, from which it can be observed that the regression line fails to pass through the coordinate origin. This indicates that intraparticle diffusion and liquid film diffusion cannot individually explain the diffusion of tetracycline from the solution to the adsorbent. Therefore, both intraparticle diffusion and liquid film diffusion control the rate at which tetracycline is transported into the adsorbent [40]. During the initial stage for all initial concentrations, the adsorption rate is relatively fast, which is due to liquid film diffusion. In the second stage, the adsorption rate slows down, as intraparticle diffusion into macropores becomes the primary factor limiting the adsorption rate, resulting in a steeper slope of the fitted line in the plot. With increasing time, the second fitted line in the plot becomes relatively flat, indicating a further slowdown in tetracycline diffusion. At this stage, the diffusion of tetracycline is restricted by mesopores and micropores [41].

The experimental data for the isotherm models are summarized in Table S2. The Freundlich model, with its fitted curve R^2^ value closer to 1, best describes the adsorption in this study onto the biochar. An n value greater than 1 in the Freundlich model proves that the modified biochar has a higher adsorption rate at low tetracycline concentrations. The Freundlich isotherm model assumes that adsorption is multilayer, while the Langmuir model assumes adsorption occurs in a monolayer manner. Therefore, tetracycline is adsorbed in a multilayer fashion onto the modified biochar prepared from KCl-ZnCl_2_ salt and bamboo powder under high-temperature pyrolysis.

### 3.4. Cost-Effectiveness Analysis

#### 3.4.1. Cycling Performance Test of the Molten Salt System

**Figure 7:**
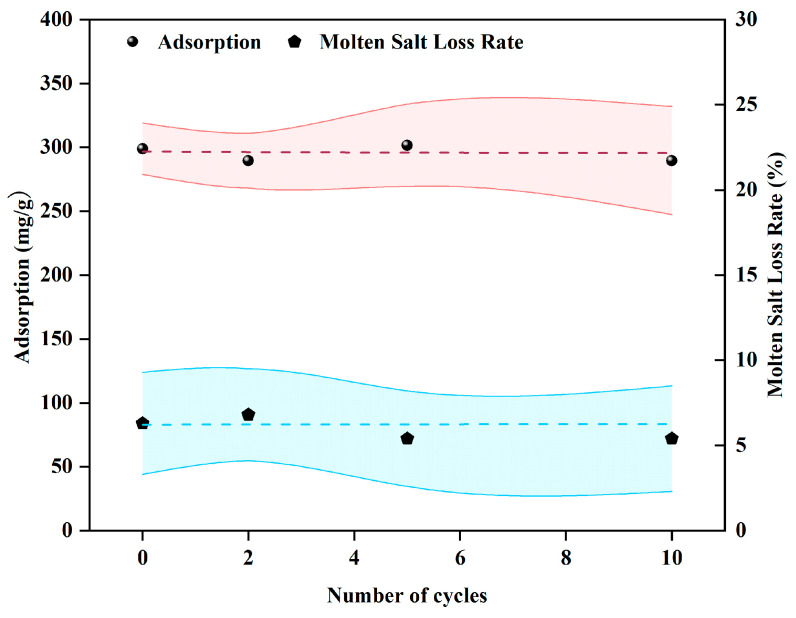
Relationship between the number of salt cycle.

The plot of tetracycline adsorption capacity versus the number of KCl-ZnCl_2_ salt cycles and the schematic diagram of the salt recycling process are presented in Figure 7 and Figure S6, respectively. As shown in Figure 7, with an increasing number of KCl-ZnCl_2_ salt recycling cycles, the adsorption capacity of the prepared MSBC900 exhibits increased oscillation amplitude around 300 mg/g. When the KCl-ZnCl_2_ salt undergoes 10 cycles, the tetracycline adsorption capacity of the modified biochar is 289.7 mg/g, which is 96.6% of the adsorption capacity of the modified biochar prepared using fresh salt. The increase in oscillation amplitude may be attributed to variations in the proportion of KCl to ZnCl_2_ in the recovered mixed salt. The recovery rate of the KCl-ZnCl_2_ salt ranges from 90% to 95%. Furthermore, the molten salt can form relatively large salt clusters, promoting phase separation between the salt and carbon phases, thereby facilitating salt recovery. Consequently, zinc chloride demonstrates favorable recyclability.

#### 3.4.2. Cost-Effectiveness Analysis of Bamboo-Based Biochar Preparation

The yields of the two biochar, MSBC800 and MSBC900, are 35.6% and 24.1%, respectively. Due to their differing adsorption performance and yields, a comparative cost analysis was conducted for biochar prepared under these two process conditions. The initial preparation cost includes the expenses for purchasing bamboo powder, zinc chloride, potassium chloride, and electricity consumption. Taking the pyrolysis of 100 kg of bamboo powder as an example, the material cost of the chemical activating agents for the initial preparation of MSBC900 and MSBC800 is as high as 12,500 CNY, accounting for 97.99% and 98.05% of the total cost, respectively. Consequently, the unit prices of MSBC900 and MSBC800 reach 529.3 CNY per kilogram and 363.2 CNY per kilogram, respectively. Therefore, the cost of chemical activating agents cannot be overlooked. However, reducing the dosage of chemical activating agents would lead to a decline in their catalytic effect, and the uncatalyzed portions would result in decreased overall stability of the biochar’s adsorption performance.

This experimental study found that activated carbon, prepared using recycled KCl-ZnCl_2,_ still exhibits favorable adsorption performance with no declining trend. Therefore, recycling KCl-ZnCl_2_ is currently the most effective method for cost reduction. From the second to the eleventh preparation, the costs of bamboo powder and electricity for each batch, along with replenishing 5–10% of the KCl-ZnCl_2_ mass to compensate for losses, are required. Under these conditions, the unit production costs of MSBC900 and MSBC800 are reduced to 40.53 CNY per kilogram and 27.83 CNY per kilogram, respectively, and the average cost decreases with an increasing number of recycling cycles. Thus, recycling KCl-ZnCl_2_ for the preparation of modified biochar can significantly lower costs, making it economically feasible.

**Figure 8:**
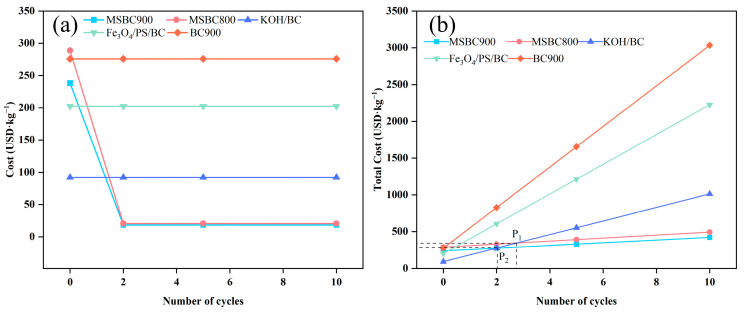
(**a**) Relationship between the number of salt cycles and the cost per cycle; (**b**) Relationship between the number of salt cycles and the cumulative total cost.

As shown in Table S3 and Figure 8, with an increase in the number of cycles, the costs of the modified biochars MSBC800 and MSBC900 are 20.58 and 18.25 USD per kilogram, respectively, which are significantly lower than those of biochars prepared via other methods. From Figure 8b, it can be observed that the total cost of preparing biochar for MSBC800 and MSBC900 in the third cycle is lower than the total cost using KOH-catalyzed preparation, indicating that recycling KCl-ZnCl_2_ can achieve the minimum total cost within a relatively short timeframe. The small vertical coordinate of point P, where the rays representing the total costs intersect, suggests a significant difference in the total costs between the two methods. When the vertical coordinate of point P is large, the unit costs of the two preparation methods are similar. The average cost after ten recycling cycles was used to estimate the expense required to remove one kilogram of tetracycline. The costs for tetracycline adsorption by MSBC900 and MSBC800 are 38.3 USD per kilogram and 46.4 USD per kilogram, respectively. In comparison, the cost ranges for removing tetracycline using other chemically modified biochars and widely used biochar-based advanced oxidation processes are 77.9 to 1791.7 USD per kilogram [42]. This demonstrates that modified biochar prepared using recycled KCl-ZnCl_2_ exhibits superior cost-effectiveness in removing tetracycline from wastewater.

## 4. Conclusions

This study reports a low-cost preparation method for high-performance bamboo-based biochar used for tetracycline removal. The modified biochar prepared by this method possesses a greater number of oxygen-containing functional groups such as carbonyl and C–O bonds, features abundant microporous and mesoporous structures, a high specific surface area, and achieves a tetracycline adsorption capacity of 298.93 mg/g. During pyrolysis, the KCl-ZnCl_2_ salt catalyzes dehydration reactions and exerts an etching effect on the bamboo powder. This process, synergized with the enhanced heat transfer of the molten salt system, promotes pore-forming activity in the biochar, creating a porous structure that consequently improves its tetracycline adsorption performance. Investigation of the process parameters revealed that an excess of adsorbent provides surplus adsorption sites, which enhances the tetracycline removal rate of the modified biochar. Within the molten salt system, increasing the activation temperature enables a leap-like improvement in the adsorption performance of the modified biochar. This enhancement occurs by altering the pore size, pore structure, as well as the types and quantities of functional groups, thereby influencing its adsorption properties. The pre-oxidation process contributes to an increase in active sites on the modified biochar. These active sites enhance the adsorption of tetracycline through van der Waals forces or π-π interactions. The order of fit for the kinetic models is Elovich > PSO > PFO. The adsorption of tetracycline is primarily driven and controlled by physisorption, including intraparticle diffusion and liquid film diffusion. The Freundlich isotherm was proven to be the most suitable, suggesting the adsorbent surface is heterogeneous. Notably, the molten salt system demonstrated excellent reusability in cycling tests, simultaneously achieving environmental friendliness and high cost-effectiveness, indicating promising potential for industrial application. Therefore, modified bamboo-based biochar exhibits significant prospects for applications in removing pollutants such as tetracycline and purifying water sources, owing to its low cost, high tetracycline adsorption performance, and environmentally friendly characteristics.

## Figures and Tables

**Table 1:** The textural properties of biochar and Modified biochar.

Sample	BET (m^2^ g^−1^)	Total Pore Volume (cm^3^ g^−1^)	Micropore Volume (cm^3^ g^−1^)	Average Pore Width (nm)
MSBC900	1305.91	0.944	0.430	1.46
MSBC800	879.465	0.526	0.330	1.18
MSBC700	412.90	0.258	0.160	1.01
MSBC600	458.01	0.263	0.177	0.96
BC900	876.86	0.428	0.335	0.86
BC800	594.07	0.285	0.227	0.79
BC700	438.97	0.240	0.168	0.86
BC600	291.81	0.196	0.110	0.99

**Table 2:** Kinetic parameters of tetracycline adsorption on biochar.

Initial Tetracycline Conc. (mg/L)	40	60	80	80 ^a^
Pseudo first order				
k_1_	0.0979	0.1171	0.1367	0.1469
q_e_ (mg/g)	108.80	163.94	201.35	267.87
R^2^	0.8868	0.9491	0.9591	0.9354
MSE	100.95	98.33	118.04	336.29
Pseudo second order				
k_2_	0.0013	0.0012	0.0012	0.0009
q_e_ (mg/g)	117.19	173.76	211.89	283.19
R^2^	0.9619	0.9903	0.9915	0.9780
MSE	33.96	18.69	24.43	114.29
Elovich				
α	988.93	15,667.27	114,123.44	89,746.35
β	0.0847	0.0720	0.0680	0.0487
R^2^	0.9941	0.9961	0.9970	0.9931
MSE	5.24	7.49	8.52	35.87

^a^ Data measured for purchased industrial activated carbon.

## Data Availability

The original contributions presented in this study are included in the article/Supplementary Material. Further inquiries can be directed to the corresponding authors.

## Supplementary Materials

The following supporting information can be downloaded at: https://www.mdpi.com/article/10.3390/ma19071457/s1, Table S1: Crystallite parameters of chars obtained from XRD patterns. Table S2: The adsorption isotherms and the R^2^ values. Table S3: Cost-benefit analysis of different biochar methods for tetracycline removal in wastewater. Figure S1: Formation mechanism of biochar porous structure: a schematic illustration. Figure S2: Adsorbent dose versus removal efficiency and adsorption capacity. Figure S3: Activation temperature versus adsorption capacity. Figure S4: Adsorption capacity and textural properties: (a) BET, (b) Tolal pore volume, (c) Micropore volume, (d) Average pore width. Figure S5: Activation temperature versus adsorption capacity. Figure S6: Schematic of biochar synthesis via salt cycling.
