# Supplementary material for: One-Step Synthesis of Bamboo Biochar for Efficiency Adsorption of Tetracycline: Characterization, Kinetics and Cost–Benefit Analysis
Source: Materials (Basel). 2026 Apr 5;19(7):1457. doi: 10.3390/ma19071457 (PMC13074597; doi:10.3390/ma19071457)
Supplement: Supplementary file 1 [file materials-19-01457-s001.zip › materials-4219296-supplementary.pdf]

Article

One-Step Synthesis of Bamboo Biochar for Efficiency Adsorption of Tetracycline: Characterization, Kinetics and Cost–Benefit Analysis

Qi Liao [1], Chengyang Cao [1,*], Qiming Zhang [1], Pei Jia [1,*] and Lu Dong [2]

[1] School of Resource & Safety Engineering, Wuhan Institute of Technology, Wuhan 430074, China; w1980016406@163.com (Q.L.); zhangqiming2736@163.com (Q.Z.)

[2] State Key Laboratory of Coal Combustion, Huazhong University of Science and Technology,
Wuhan 430074, China; ludong@hust.edu.cn

*Correspondence: ccy9311@wit.edu.cn (C.C.); 04001074@wit.edu.cn (P.J.)

Table S1 Crystallite parameters of chars obtained from XRD patterns.

Sample	d_{002}(nm)	Lc (nm)	Lα(nm)
BC600	0.3862	0.0134	0.0063
BC700	0.3856	0.0155	0.0353
BC800	0.3741	0.0150	0.0596
BC900	0.3823	0.0152	0.1052
MSBC600	0.3923	0.0159	0.0066
MSBC700	0.3829	0.0166	0.0235
MSBC800	0.3875	0.0187	0.0435
MSBC900	0.3929	0.0149	0.0815

Table S2 The adsorption isotherms and the R^2 values.

Models	Parameters			R^2	MSE
Langmuir	k_L (l mg^{-1})	q_m (mg/g)		0.9217	247.2113
	0.0217	445.6065			
Freundlich	k_f (l g^{-1})	n		0.9828	72.2763
	60.3463	1.9981			
Sips	k_s (l mg^{-1})	q_m (mg/g)	s	0.9959	106.3824
	0.0021	2027.1048	0.5862		

Table S3 Cost-benefit analysis of different biochar methods for tetracycline removal in wastewater.

Preparation method	Cost ($USD \cdot kg^{-1}$)	Preparation method	Cost ($USD \cdot kg^{-1}$)
Aerated SSB	49.8	g-C_3N_4/BiOBr/BC	1722.4
Aerated PSB	51.2	CeO_2/Fe_3O_4/BC	1740.1
Aerated PWB	54.7	Calcium/BC	1791.7
Aerated RSB	76.4	PDS/BC	78.9
WFB	67.4	Fe_3O_4/PS/BC	202.4
ALB	153.7	Montmorillonite/BC	885.6
Phosphoric/BC	77.9	Silver nanoparticles/BC	1058.7
$Fe_2(SO_4)_3$/BC	79.4	MSBC800	20.6
KOH/BC	92.1	MSBC900	18.3
SiO_2/BC	901.3	BC900	279.9
H_2O_2/BC	1444.1		

PSB: Peanut shell biochar, RSB: Rice straw biochar, SSB: Sewage sludge biochar, PWB: Putrescible waste biochar. WFB: Waste fiberboard biochar, ALB: Auricularia residue biochar, BC: biochar, PDS: Peroxydisulfate, PS: Persulfate.

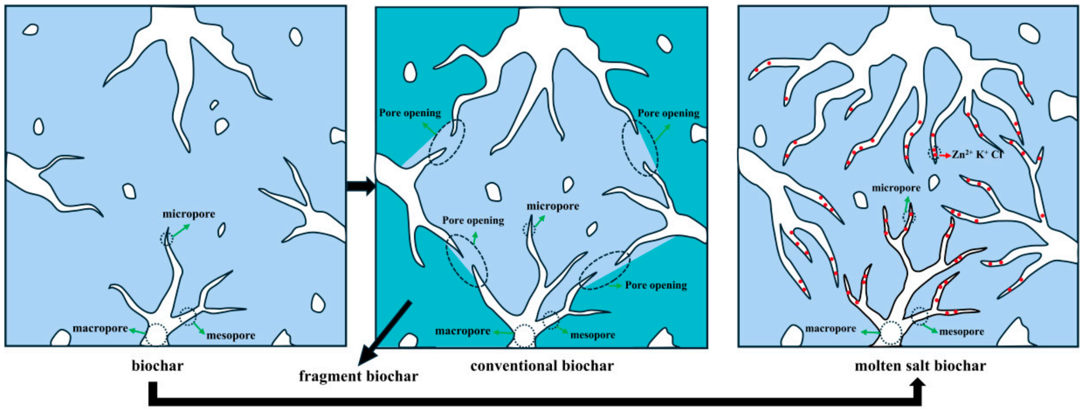

Figure. S1. Formation mechanism of biochar porous structure: a schematic illustration.

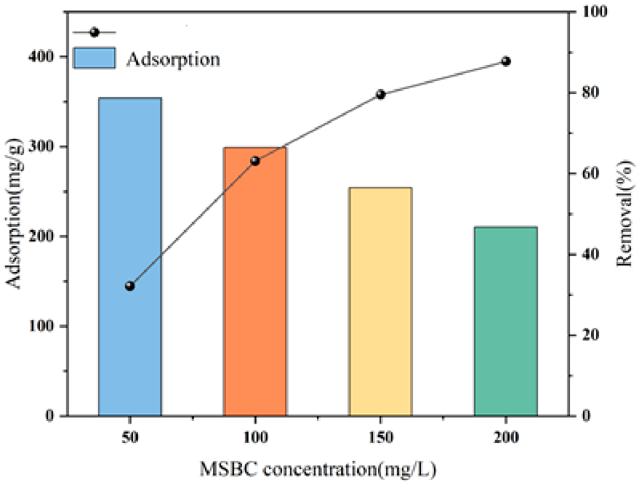

Figure. S2. Adsorbent dose versus removal efficiency and adsorption capacity.

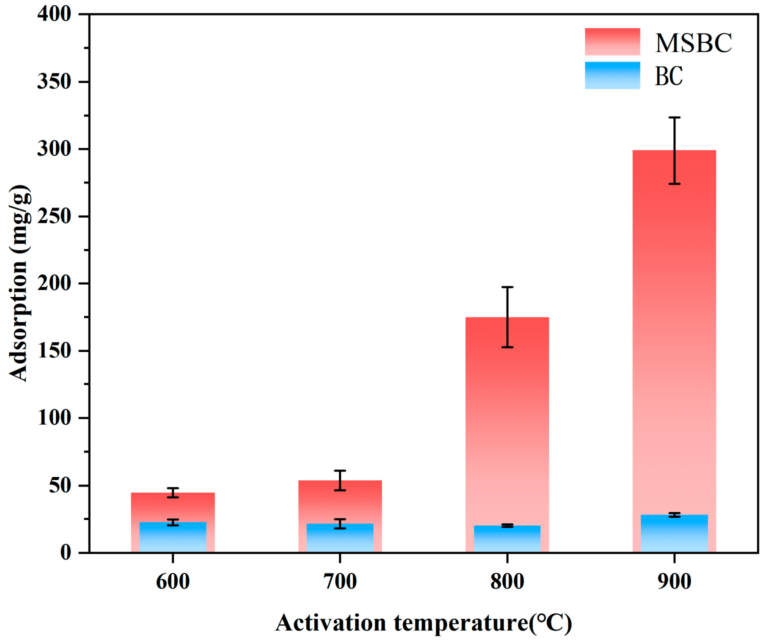

Figure. S3. Activation temperature versus adsorption capacity

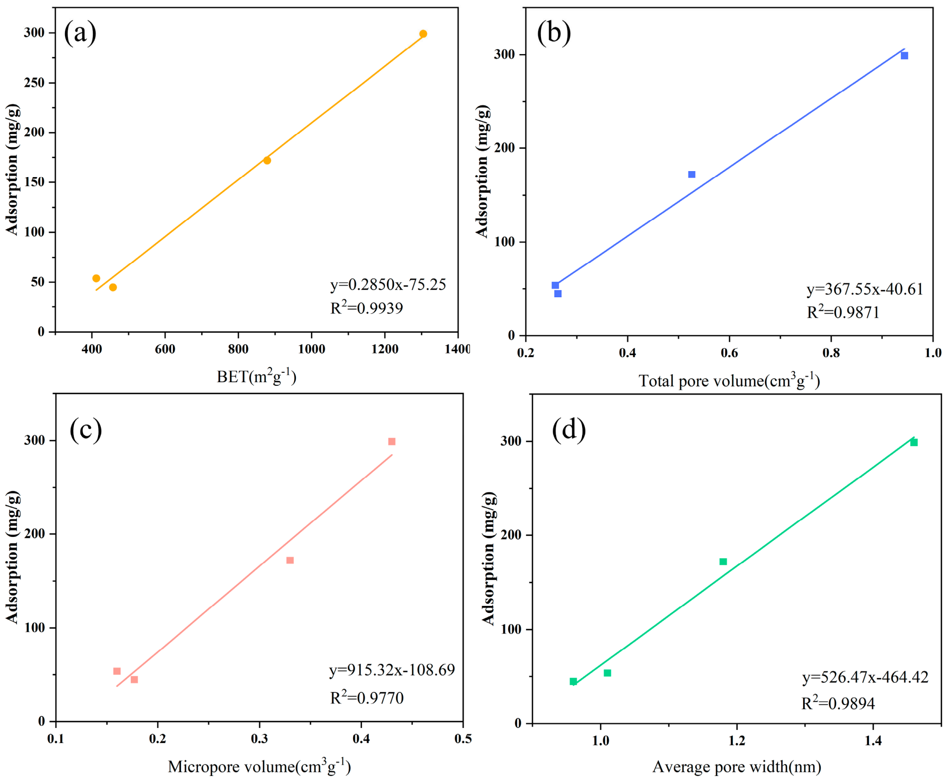

Figure. S4. Adsorption capacity and textural properties: (a) BET, (b) Tolal pore volume, (c)Micropore volume, (d) Average pore width.

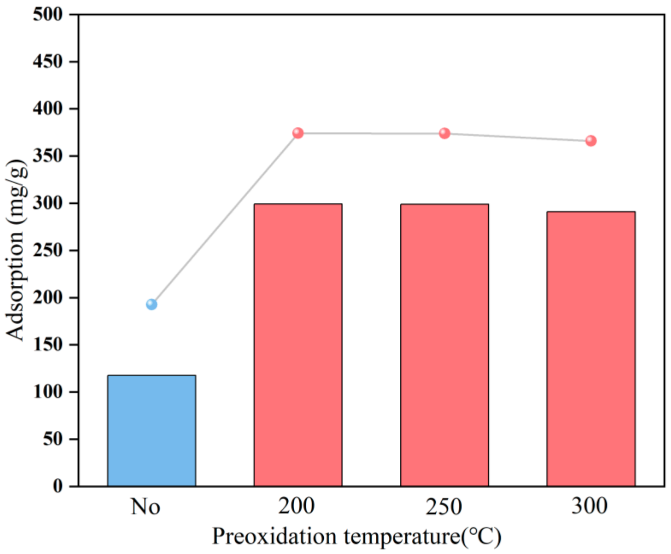

Figure. S5. Activation temperature versus adsorption capacity

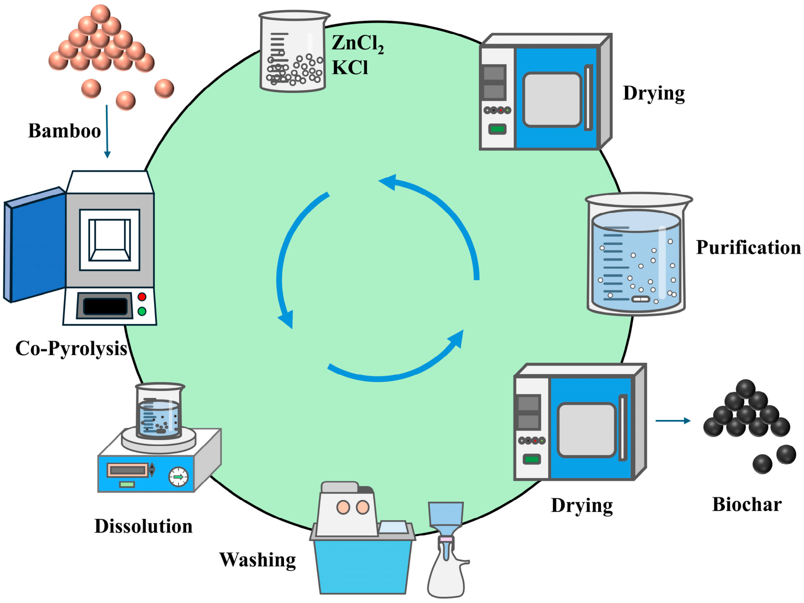

Figure. S6. Schematic of biochar synthesis via salt cycling.
